# Safety and efficacy of continuous cefazolin infusion via peripheral intravenous catheter (PIVC) for outpatient therapy: a brief report

**DOI:** 10.1093/jacamr/dlaf169

**Published:** 2025-09-23

**Authors:** Hubert Chan, Solly Joseph, Fiona Tran, Ravindra Dotel, Vincent J J Ngian, Bin S Ong

**Affiliations:** Bankstown-Lidcombe Hospital, Bankstown, Sydney, New South Wales, Australia; Department of Ambulatory Care Medicine, Bankstown-Lidcombe Hospital, Bankstown, Sydney, New South Wales, Australia; Bankstown-Lidcombe Hospital, Bankstown, Sydney, New South Wales, Australia; Department of Ambulatory Care Medicine, Bankstown-Lidcombe Hospital, Bankstown, Sydney, New South Wales, Australia; Bankstown-Lidcombe Hospital, Bankstown, Sydney, New South Wales, Australia; Department of Ambulatory Care Medicine, Bankstown-Lidcombe Hospital, Bankstown, Sydney, New South Wales, Australia; Department of Geriatrics, Bankstown-Lidcombe Hospital, Bankstown, Sydney, New South Wales, Australia; School of Clinical Medicine, University of New South Wales, Sydney, New South Wales, Australia; Bankstown-Lidcombe Hospital, Bankstown, Sydney, New South Wales, Australia; Department of Infectious Diseases, Bankstown-Lidcombe Hospital, Bankstown, Sydney, New South Wales, Australia; Bankstown-Lidcombe Hospital, Bankstown, Sydney, New South Wales, Australia; Department of Ambulatory Care Medicine, Bankstown-Lidcombe Hospital, Bankstown, Sydney, New South Wales, Australia; Department of Geriatrics, Bankstown-Lidcombe Hospital, Bankstown, Sydney, New South Wales, Australia; School of Clinical Medicine, University of New South Wales, Sydney, New South Wales, Australia; Bankstown-Lidcombe Hospital, Bankstown, Sydney, New South Wales, Australia; Department of Ambulatory Care Medicine, Bankstown-Lidcombe Hospital, Bankstown, Sydney, New South Wales, Australia; Department of Geriatrics, Bankstown-Lidcombe Hospital, Bankstown, Sydney, New South Wales, Australia; School of Clinical Medicine, University of New South Wales, Sydney, New South Wales, Australia

## Abstract

**Background:**

Cefazolin elastomeric infusers are commonly used in outpatient settings with peripherally inserted central catheters (PICCs) or mid-line catheters to treat various infections. However, data on the safety of cefazolin elastomeric infusers administered via a peripheral IV catheter (PIVC) are limited.

**Objectives:**

To assess the safety and complications of outpatient cefazolin elastomeric infusers via PIVC.

**Methods:**

A retrospective case series study was conducted, reviewing patients treated with cefazolin elastomeric infuser via PIVC between January 2018 and December 2022 in our institution. The primary outcomes assessed were PIVC-related complications using visual infusion phlebitis (VIP) score, including catheter-related bacteraemia and readmissions within 30 days.

**Results:**

A total of 415 patients aged ≥18 years were treated with cefazolin elastomeric infuser through PIVC during the 5 year study period. On average, each patient required two PIVCs during outpatient treatment, amounting to 870 PIVCs in total. Of these, 85% (*n* = 352) of patients received cefazolin 6 g daily. The majority were treated for cellulitis (83%) with a median duration of 5 days. No complications (VIP score = 0) were observed in 88.3% (*n* = 768) of catheters. The incidence of VIP scores of 1, 2 and ≥3 was 8.9% (*n* = 37), 4.1% (*n* = 17) and 0% (*n* = 0), respectively. There were no statistically significant differences in age, sex, comorbidity or diagnosis between participants with a VIP score of 0 and those with a VIP score of ≥1. The incidence of complications including catheter leakage, blockage and dislodgement was low (2.3%, 1.6% and 2%, respectively).

**Conclusions:**

Outpatient cefazolin elastomeric infusers administered via PIVC were well tolerated and safe.

## Introduction

Out-of-hospital antimicrobial therapy administered via outpatient parenteral antimicrobial therapy (OPAT) or hospital-in-the-home (HITH) programmes can provide cost-effective therapy for a variety of infections.^1,2^ IDSA’s clinical practice guidelines recommend a peripherally inserted central catheter (PICC) or a mid-line catheter for vascular access to provide continuous antimicrobial infusion therapy in the OPAT setting.^3^ These require specially trained staff for insertion and may cause venous thrombosis and pneumothorax.^4,5,6^ In contrast, a peripheral IV catheter (PIVC) requires minimal training, and lower maintenance requirements and cost.^4,7^

Cefazolin is the most commonly used antibiotic in the OPAT setting. Soft-tissue infections requiring community-based parenteral antimicrobial therapy receive either cefazolin 2 g twice daily or 2 g IV once daily with oral probenecid.^8,9,10^ Patients requiring higher doses of cefazolin are generally treated as inpatients in a hospital or receive continuous antibiotic infusion via a PICC or mid-line catheter in the OPAT setting.^3^

Elastomeric antimicrobial infusers, lightweight disposable devices, provide continuous infusion over 24 h.^11^ Current guidelines recommend elastomeric infuser pumps to be connected to PICCs or mid-line catheters as vascular access.^3^ An alternative would be continuous antibiotic infusion via an elastomeric infuser pump through a PIVC but this is rarely utilized.^12^ There are concerns that antibiotic infuser administration through PIVCs may pose a risk of line infection, bacteraemia, extravasation, dislodgement and risk of phlebitis in the outpatient setting, where there is less supervision.^13,14,15^

The aim of the study was to assess the safety, complications and efficacy of cefazolin infuser administration through a PIVC in infections requiring cefazolin in the out-of-hospital setting.

## Method

This was a single-centre outpatient descriptive retrospective case series conducted in the OPAT/HITH programme of the Ambulatory Care Unit in Bankstown-Lidcombe Hospital, New South Wales, Australia from January 2018 to December 2022. The study was approved by the South-Western Sydney Local Health District (SWSLHD) Human Research Ethics Committee. The ethics committee assessed the research as a low-risk study with no identifiable information, therefore allowing a waiver of informed consent. Data were collected through electronic medical records.

All patients aged ≥18 years who received IV cefazolin via an elastomeric infuser administered through a PIVC in the OPAT/HITH programme were included. The cases were referred from the emergency department, GPs and inpatient hospital teams. Exclusion criteria were allergy to cephalosporins, known resistance to cefazolin from antibiotic susceptibility results, severe sepsis, inability to care for themselves or lack of carer, and history of IV drug use. Data on patient characteristics collected included age, sex, BMI, renal function, prior antibiotics used, type of infections and Charlson comorbidity index.^12,15,16^

A 22-gauge non-integrated PIVC was used in all patients.^7^ Nursing staff provided instruction on managing the elastomeric infusers at home. Patients undergoing treatment were reviewed daily and a new infuser was connected in the ambulatory care unit. Patients receiving 6 g IV cefazolin elastomeric infusion over 24 h had estimated glomerular filtration rates (eGFRs) of >40 mL/min/1.73 m^2^. A higher dose of cefazolin, 8 g IV, was used in patients with a BMI of >50 kg/m^2^.^17^ Each infuser contained 240 mL infusing at 10 mL/h over 24 h.

PIVC sites were assessed daily by trained nurses using the visual infusion phlebitis (VIP) score for complications including erythema, tenderness, extravasation, induration and thrombophlebitis. VIP score is recommended in the international Infusion Nursing Standards of Practice to assess phlebitis.^18^ VIP score ranges from 0 to 5, in increasing order of inflammation severity. PIVC site infection, vein thrombosis and bacteraemia were recorded. Reasons for PIVC removal included routine change of PIVC after 3 days or cessation of IV treatment, leaking of catheter, blockage of PIVC, dislodgement, PICC line insertion and VIP score of 1 or more.^19^ PIVC dwell time and number of PIVCs inserted for each patient were recorded.

The primary outcomes were PIVC-related complications including VIP score, PIVC site infection, bacteraemia and readmission due to PIVC complication within 30 days. Secondary outcomes assessed were readmission for recurrent infection within 30 days and mortality.

Descriptive statistics were used to summarize patient demographics, clinical characteristics and outcomes. Continuous variables were presented as mean and standard deviation (SD) or median and IQR, while categorical variables were reported as frequencies and percentages. To identify factors associated with phlebitis, patient characteristics were compared between those with a VIP score of 0 and those with a VIP score of ≥1. The independent samples *t*-test was used for continuous variables, and Pearson’s chi-squared test was used for categorical variables. A *P* value of <0.05 was considered statistically significant for all analyses.

## Results

A total of 415 participants were treated with cefazolin via elastomeric infuser during the 5 year study period (Jan 2018—Dec 2022). The patient characteristics are shown in Table 1. The mean BMI in this study population was in the obesity range: 33.6 (±9.4) kg/m^2^. The most common soft-tissue infection was cellulitis (84%; *n* = 347). Apart from two patients with a BMI of >50  kg/m^2^, who received 8 g cefazolin, the majority received 6 g IV cefazolin (85%; *n* = 353). The remaining patients received 4 g IV cefazolin (Table 1).

**Table 1.** VIP score and patient characteristics

Characteristics	Overall *n* = 415	VIP score = 0 *n* = 360	VIP score ≥ 1 *n* = 55	*P* value^a^
Age (years), mean (SD)	54.8 (17.9)	54.6 (17.6)	55.6 (20.2)	0.5
Sex, *n* (%)				0.04
Female	127 (31)	104 (28.9)	23 (42.6)	
Male	288 (69)	256 (71.1)	31 (57.4)	
Diagnosis, *n* (%)				0.4
Joint or bone infection^b^	8 (1.9)	7% (1.9)	1 (1.9)	
Wound infection^c^	53 (12.7)	45 (13)	8 (15)	
Cellulitis	347 (83)	303 (84)	43 (80)	
Other infection^d^	7 (1.6)	5 (1.4)	2 (3.7)	
Change of therapy, *n* (%)^e^	36 (8.7)			
Readmission, *n* (%)^f^	15 (3.6)			
Prior antibiotics, *n* (%)	308 (74)			
Comorbidity, *n* (%)				
IHD	43 (10)	37 (10)	6 (11)	0.9
CCF	16 (3.9)	14 (3.9)	2 (3.7)	>0.9
PVD	8 (1.9)	6 (1.7)	2 (3.7)	0.3
CVA/TIA	7 (1.7)	6 (1.7)	1 (1.9)	>0.9
COPD	59 (14)	49 (14)	10 (19)	0.3
Diabetes	92 (22)	79 (22)	13 (24)	0.7
Diabetes EOD	18 (4.3)	17 (4.7)	1 (1.9)	0.5
Malignancy	36 (8.7)	29 (8.1)	7 (13)	0.3
CTD	6 (1.4)	6 (1.7)	0 (0)	>0.9
Hypertension	156 (38)	136 (38)	20 (37)	>0.9
Ulcer/cellulitis	111 (27)	92 (26)	19 (35)	0.14
Depression	32 (7.7)	24 (6.7)	8 (15)	0.05

IHD, ischaemic heart disease; CCF, Congestive cardiac failure; PVD, peripheral vascular disease; CVA, cerebral vascular accident; TIA, transient ischaemic attack; Diabetes EOD, diabetes end organ damage; CTD, connective tissue disease.

^a^Pearson’s chi-squared test.

^b^Osteomyelitis, paronychia, tenosynovitis and bursitis.

^c^Ulcer or abscess.

^d^Other infections include bacteraemia, perichrondritis and infected sebaceous cyst.

^e^Change of therapy to piperacillin/tazobactam.

^f^Readmission within 30 days for recurrent infection.

The median duration of IV cefazolin treatment was dependent on the type of infection. For cellulitis it was 5 days (IQR 3.7). Patients with longer treatment duration, more than 20 days, were treated for ulcer/wound infection (0.7%, *n* = 3). The majority of patients had a PIVC dwell time of 3 days (42%, *n* = 370), with the longest PIVC dwell time of 5 days (1.4%, *n* = 12). Of the 415 patients, 154 (37%) required one PIVC during the treatment period. A further 134 (32%) patients required two PIVCs during their treatment course and the remaining 117 (28%) had three or more PIVCs. Of the 870 PIVCs inserted, 88.3% (*n* = 768) had a VIP score of 0 and 11.7% (*n* = 102) had a VIP score of ≥1.

Reasons for PIVC change are shown in Table 2. The highest severity of PIVC complication was non-infective phlebitis, with a VIP score of 2. PIVCs were re-sited with a VIP score of ≥1. In this study there were no cases of local catheter site infection or PIVC-related bacteraemia. The readmission rate for recurrent infection within 30 days was 3.6% (*n* = 15). There was no mortality within the 30 days of follow-up.

**Table 2.** Indications for PIVC change

PIVC re-site reasons	*n* (*N* = 870)	%
Routine change	583	67.0
No longer required	78	9.0
Possible phlebitis (VIP 1)	55	6.3
Early phlebitis (VIP 2)	47	5.4
Request by patient	22	2.5
Leaking	20	2.3
Dislodged	17	2.0
Admitted	15	1.7
Blocked	14	1.6
Kinked	14	1.6
PICC inserted	5	0.6

## Discussion

This study supports the safety of cefazolin elastomeric infuser via PIVC for outpatient therapy. No severe PIVC-related complications, including PIVC-related infections or bacteraemia were observed. The phlebitis rate was 11.7% (*n* = 102), which was lower compared with other reported studies of cefazolin infuser via PIVC (13.6%) or general PIVC use (19.3%).^13,14^ Other previously reported complications such as leakage and occlusion rate were 7% and 8%, respectively, whereas in our study the rates were 2.3% and 1.6%, respectively.^13^ We postulated that the lower PIVC-related complication rate may be due to daily review of PIVCs and the utilization of the forearm site in our protocol, which has been associated with lower complication rates.^15^

Age, sex, type of infection and comorbidities were not associated with an increased incidence of phlebitis in this study (Table 1). There was no documented case of hospital admission while on therapy. This may reflect the less severe cases in our study treated in the outpatient setting. In our study we found no severe PIVC-related complications requiring treatment. Patients requiring more than 10 days of antibiotics for various infections, including osteomyelitis, chronic wound infection and bacteraemia, tolerated multiple cannulations without significant complications for the duration of their therapy.

Cefazolin infusers allowed dose adjustment according to the patient characteristics such as BMI, renal function and severity of infection, with the majority of our patients receiving 6 g IV cefazolin. Patients with very high BMIs (>50 kg/m^2^) received 8 g IV cefazolin, all tolerating the higher dose of cefazolin with no increased risk of PIVC complication, renal impairment or side effects.^17^ This only accounts for a small number of patients in our study, and a larger number would be needed to assess the safety of high-dose cefazolin infuser through a PIVC.

There are limitations to consider when interpreting the results in our study. The retrospective nature of the study design was dependent on the accuracy of clinician documentation, but the main data were objectively defined. Other common parenteral antibiotics used in the outpatient setting such as ceftriaxone, vancomycin, flucloxacillin and piperacillin/tazobactam were not included in this study. We focused on cefazolin as it is the most commonly used antibiotic in our OPAT setting.^9^ A larger sample size with a multicentre study would add validity to the study results. In this study, patients were coming into the ambulatory care unit each day for infuser change and review of the PIVC site. The next stage of the study is to examine this model in the patients’ home settings.

### Conclusions

Outpatient elastomeric cefazolin infuser via PIVC was well tolerated, with no increased risk of severe PIVC-related complications. We recommend its use with close supervision and further research to validate these findings in other centres.
